# Medical Resident Awareness of Predatory Journal Practices in an International Medical Education System

**DOI:** 10.1080/10872981.2022.2139169

**Published:** 2022-10-21

**Authors:** Halah Ibrahim, Shahad Abasaeed Elhag, Salma M Elnour, Sawsan Abdel-Razig, Thana Harhara, Satish Chandrasekhar Nair

**Affiliations:** a Khalifa University College of Medicine and Health Sciences, Abu Dhabi, United Arab Emirates; b Sheikh Khalifa Medical City, Abu Dhabi, United Arab Emirates; c Cleveland Clinic Abu Dhabi, Abu Dhabi, United Arab Emirates; d Academic Affairs Department, Tawam Hospital, Al Ain, United Arab Emirates

**Keywords:** Predatory journals, predatory publishing, medical education, research methodology

## Abstract

**Introduction:**

Learning research methodology is increasingly becoming an essential part of graduate medical education worldwide, with many regulatory and accreditation bodies requiring residents to participate in scholarship. Research methodology workshops have become a standard part of medical curricula; however, there is limited data on how much training on journal selection and the publication process trainees receive. The alarming growth of predatory journals has made it increasingly difficult for researchers, especially trainees and early career physicians, to distinguish these publications from reputable journals. The purpose of this study is to assess the knowledge of reputable and predatory publishing practices amongst medical trainees in an international medical education setting in the United Arab Emirates.

**Methods:**

A survey on credible journal practices based on the ‘Think. Check. Submit’ initiative was sent to all graduate medical education trainees at two large academic medical centers in Abu Dhabi, United Arab Emirates. Descriptive statistics were used to tabulate variable frequencies.

**Results:**

Over half of the 160 respondents reported receiving prior research methodology training and 42.5% had at least one publication. The majority of the trainees selected impact factor and the quality of the peer-review process as characteristics of reputable journals. Ambiguous editorial board and rapid publication process were recognized as characteristics of predatory journals by >65% of trainees, however, 95% of all trainees were unaware of Beall’s list or other resources to help select a journal for publication. 15.2% of trainees who received unsolicited emails from publishers submitted their manuscripts to the unfamiliar journals, citing peer recommendation and pressure to publish from their training programs as reasons.

**Conclusion:**

Trainees in the United Arab Emirates were mostly unaware of reputable publication practices and are vulnerable to publishing in predatory journals. Policy and educational reform are necessary to maintain the credibility and integrity of the scientific process.

## Introduction

High quality research drives evidence-based guidelines and forms the foundation for medical education and clinical practice. Studies have shown that resident participation in research encourages careers in academic medicine, enhances clinical reasoning, promotes evidence-based practices, and ultimately improves patient care [1]. Learning research methodology is increasingly becoming an important part of graduate medical education worldwide [2]. Moreover, many regulatory bodies and accreditation agencies require residents to participate in scholarly activities, making research involvement and publications in medical journals an expected component of trainee portfolios and a key determinant of career progression. As a result, residency programs have incorporated research methodology workshops into their curricula, with many requiring residents to publish prior to completing training [2]. Despite the emphasis on how to effectively conduct research, there is limited data on how much information trainees receive on the publication process and how to select the right journals for their studies. This is complicated by the rapid transformation of scholarly publishing over the past two decades, and most notably, by the introduction of open access journals and the emergence of predatory journals and publishers.

In the early 2000s, initiatives, including the Budapest Open Access Initiative and the Berlin Declaration on Open Access to Knowledge in the Sciences and Humanities, sought to accelerate dissemination and broaden visibility of scientific work through open access publishing [3,4]. In the open access model, authors pay an article processing charge for journals to make their articles freely available online, thereby making research more transparent and accessible [4]. This model was soon exploited by unscholarly open access publishers that subverted the peer review publication system for financial gain [5]. Jeffrey Beall, a librarian at the University of Colorado in Denver, was the first to raise concern about these practices and coined the term ‘predatory’ journals and publishers [6]. Predatory journals are counterfeit productions that take advantage of open access publishing models to gain profit through publication fees [6,7]. They target authors primarily through mass emails and aggressive marketing that promise a speedy publishing process and an almost certain approval for publication [7,8]. While there is recognition that predatory publishing threatens the integrity of research and scholarship, identifying and keeping up with this rapidly growing phenomenon has challenged the biomedical community [6–8]. Researchers have attempted to describe the characteristics of predatory journals, resulting in several definitions [6–9]. Cobey and colleagues conducted a scoping review of the literature on predatory journals, and noted a lack of a consensus definition and substantial heterogeneity in the descriptions of predatory journals [10]. In 2019, an international group of researchers, academicians, policy makers, journal editors and publishers, and patient representatives convened and developed a consensus definition, which identified four main characteristics of predatory journals or publishers: ‘Predatory journals and publishers are entities that prioritize self-interest at the expense of scholarship and are characterized by false or misleading information, deviation from best editorial/publication practices, lack of transparency, and/or use of aggressive and indiscriminate solicitation practices’ [7,9]. Elaborate and deceitful strategies employed by predatory journals, such as using websites that mimic legitimate journals (hijacked journals), citing credible researchers as editorial board members without their consent, and reporting misleading metrics or false impact factors, have posed real challenges [11,12]. Recent studies estimate that approximately 8000 predatory journals currently operate [12]. Individual and organizational efforts have attempted to curtail these fraudulent practices, including the Beall list (aimed at identifying and publicly naming predatory journals) and the Directory of Open Access Journals (DOAJ) (which provide certification of open access journals) [13,14]. Currently, there are over 90 checklists available to help authors identify predatory journals [7]. The checklist approach, however, has uncertain validity and utility, and is fraught with controversy [15–18]. Researchers disagree on the weight and evaluation of inclusion criteria and have noted that the checklists are inconsistent and often not based on research evidence [15]. Several studies have found discrepancies between lists, as well as potential bias and conflicts of interest in list development, negatively impacting credibility and creating confusion for authors [15–19]. In addition, newer journals may share some of the characteristics of predatory journals and have been mistakenly labeled as illegitimate [20]. Koerber and colleagues conducted a qualitative content analysis of the inclusion and exclusion criteria of scholarly publishing journal checklists and concluded that all lists were incomplete and outdated, primarily due to the rapidly changing tactics employed by predatory publishers [20].

As predatory publishing has become too complex to be captured by individual lists, researchers are beginning to explore the efficacy of awareness and educational campaigns on reputable and illegitimate journal practices [21]. Studies in the USA have shown a lack of knowledge and awareness of predatory publishing practices among physicians at all career levels, and especially among medical students and trainees [22]. It has also been reported that researchers that publish in predatory journals tend to be young, inexperienced authors from developing countries [22,23]. With the rising popularity of open access as a publication model and the continued lack of clarity between predatory and legitimate open access processes [24], authors in the global context may be particularly vulnerable to predatory publication practices. The purpose of this study is to assess the knowledge of reputable and predatory publishing practices amongst medical trainees in an international medical education and research environment in the United Arab Emirates (UAE).

## Methods

We conducted a cross-sectional survey of medical trainees at two large academic medical centers in the UAE between November 2021 and January 2022. We used the Strengthening the Reporting of Observational Studies in Epidemiology (STROBE) guidelines for reporting cross-sectional studies to report our findings [25].

### Setting and Population

Participants included all graduate medical education trainees (interns, residents and fellows) at two large academic medical centers in Abu Dhabi, UAE. The UAE is a small nation in the Middle East that has invested heavily over the past two decades in its healthcare and medical education infrastructure [26]. In 2012, all residency training programs in Abu Dhabi, the capital and most populous emirate in the country, transitioned to competency based medical training based on a USA residency training model and received accreditation by the Accreditation Council for Graduate Medical Education International (ACGME-I) [26,27]. In compliance with ACGME-I requirements, trainees and teaching faculty are expected to participate in scholarly activity, and several initiatives have been developed to improve resident research productivity [28,29]. This study was approved by the Sheikh Khalifa Medical City Institutional Review Board [RS-731] and the Cleveland Clinic Abu Dhabi Research Ethics Committee [B-2021-002]..

### Survey Development

Survey questions were developed after a comprehensive review of the literature on credible journal practices and based on the checklist of the ‘Think. Check. Submit’ initiative, an international strategy that provides guidelines for reputable journal selection. The project aims to uphold research integrity by promoting credible research and publications [30]. The instrument was reviewed by the Director of Clinical Research at one of the participating hospitals and then piloted for clarity and comprehension on ten residents in an affiliated hospital that was not included in this study. Minor contextual changes were made based on their feedback. The final survey consists of four sections. Following demographics, respondents were asked questions regarding their publication history. The next section explored factors contributing to respondents’ selection of journals for publication. Responses were presented as a four point likert scale, from ‘not at all important ’ to ‘very important.’ The final set of questions focused on respondents’ knowledge of credible and predatory journal practices. In this section, an ‘I don’t know’ option was available.

### Data Collection

Trainee emails were obtained from each hospital’s education department. In November 2021, each trainee received an email invitation with an individual link to an online survey. E-mail reminders were sent every two weeks, with a total of three reminders. The emails described the purpose of the study and explained that participation was voluntary and anonymous. No identifying data was collected and no incentives were offered. Consent to participate in the study was indicated by the completion and return of the survey.

### Data Analysis

Data collected were analyzed using SPSS (IBM, Chicago, Version 25). Descriptive statistics to determine the frequencies of the various variables were used and tabulated as percent (%) and actual numbers (n). Chi-square test was employed to assess the correlation between the various variables and demographics. P value <0.05 was considered significant.

## Results

Of 218 surveys distributed, 160 responses were received (73.4% response rate). Participant demographics are listed in Table 1. There were no significant demographic differences between responders and non-responders. Approximately 40% of the trainees had at least one publication (n = 68; 42.5%), with 60.8% of them opting for an open access journal. Most residents (>85%) reported that improving their candidacy for future residency/fellowship or job position and professional recognition were the most important reasons for participation in research activity (Table 2). This was consistent across genders and training levels (p < 0.39–0.56, data not shown).

**Table 1.** Demographics of survey respondents (N = 160).

Demographic		n (%N)
Gender	Male	44 (27.5%)
	Female	116 (72.5%)
Level of Training	Senior Residents (PGY3-4, fellows)	73 (45.6%)
	Junior Residents (Interns, PGY1-2)	87 (54.4%)
Received research training	Yes	86 (53.8%)
	No	74 (46.3%)
History of publishing	Yes	68 (42.5%)
	No	92 (57.5%)

**Table 2.** Factors contributing to trainee decision to participate in research (N = 160).

	Important	Not important
	n(%N)	n(%N)
Improve your candidacy for future residency/Followship	155 (96.9%)	5 (3.1%)
Professional Recognition	140 (87.5%)	20 (12.5%)
Requirement of institution/program	138 (86.3%)	22 (13.8%)
Adopt Evidence Based Practice	132 (82.5%)	28 (17.5%)
Peer Collaboration	117 (73.1%)	43 (26.9%)
Personal Recognition	109 (68.1%)	51 (31.9%)

Over half of the trainees (n = 86; 53.8%) reported receiving prior research methodology training. The vast majority of residents (95%), irrespective of history of research education, were not aware of Beall’s list or any resources to help select a suitable journal for publication. Factors that impacted trainee journal selection were faculty recommendation, indexing in a major database, and the publishing experiences of peers, with 89.4%, 86.2%, and 85.6% of trainees respectively selecting these three criteria as important considerations when submitting a manuscript (Table 3).

**Table 3.** Factors trainees consider when submitting manuscripts to a scientific journal (N = 160).

	Important	Not important
	n(%N)	n(%N)
Recommendation from faculty/mentor	143 (89.4%)	17 (10.6%)
Indexing in a major database	138 (86.2%)	22 (13.8%)
Publishing experiences of other colleagues	137 (85.6%)	23 (14.4%)
High-impact factor	133(83.1%)	27 (16.9%)
Speciality of journal	128 (80%)	32 (20%)
Affordable publication fee	126 (78.8%)	34 (21.3%)
Reputable editorial board	124 (77.5%)	36 (22.5%)
Open access for readers	116 (72.5%)	44 (27.5%)
Recommendation from peers	112 (70%)	48 (30%)
Rapid publication process	110 (68.7%)	50 (31.3%)
Country of publication	100 (62.5%)	60 (37.5%)

When asked to describe characteristics of reputable journals, the impact factor was the most selected feature (91.9%), followed by the quality of the peer review process (84.4%) (Table 4). Most of the trainees recognized that certain characteristics, such as an ambiguous editorial board, unprofessional journal layout, incomplete author guidelines, and rapid publication process, were associated with predatory journals (Table 5). The majority of trainees (n = 147; 91.8%) expressed interest in learning more about reputable journal practices.

**Table 4.** Characteristics of reputable journals.

	Important	Not important
	n(%N)	n(%N)
Impact factor	147 (91.9%)	13 (8.1%)
Quality of the peer-review process	135 (84.4%)	25 (15.6%)
Quality of the submission system	132 (82.5%)	28 (17.5%)
Scope of the journal	127 (79.4%)	33 (20.6%)
Reputation of the editorial board	120 (75%)	40 (25%)
Detailed author guidelines	118 (73.7%)	42 (26.3%)
Transparency of publication fees	115 (71.9%)	45 (28.1%)
Reputation of the publisher	111 (69.4%)	49 (30.6%)
Submission fee costs	104 (65%)	56 (35%)
Publication fee costs	104 (65%)	56 (35%)
Layout of the journal	84 (52.5%)	76 (47.5%)
Country of publication	84 (52.5%)	76 (47.5%)

**Table 5.** Characteristics associated with predatory journals.

	Associated	Not Associated
	n(%N)	n(%N)
Ambiguous editorial board	105 (69.1%)	47 (30.9%)
Low impact factor	103 (67.8%)	49 (32.2%)
Rapid publication process	95 (62.5%)	57 (37.5%)
Journal is located in a newly industrializing country	94 (61.8%)	58 (38.2%)
Low quality of the published article	93 (61.2%)	59 (38.9%)
Missing or incomplete author guidelines	91 (59.9%)	61 (40.1%)
High publication fees	88 (57.9%)	64 (42.1%)
Missing or incomplete author guidelines	83 (54.6%)	69 (45.4%)
Unprofessional journal layout	57 (37.5%)	95 (62.5%)

Although most residents reported familiarity with subscription (n = 134; 83.8%) and open access models (n = 136; 85%), 75% of survey participants were not familiar with the concepts of transformative journal, hijacked journal, or post publication peer review options. 28.7% (n = 46) of trainees reported receiving solicitation emails from unfamiliar journals, with 15.2% (n = 7) admitting to submitting manuscripts to the unfamiliar journal, with peer recommendations, open access for readers, and pressure from their training program to publish as the most cited reasons for submitting to the predatory journal. Training level differences were noted, with junior residents (25%) more likely than senior residents (6%) to submit their research to an unfamiliar journal (data not shown).

## Discussion

In this survey study of medical interns, residents, and fellows at large academic medical centers in the UAE, a significant proportion of trainees had received training in research (53.8%) with many having publication experience (42.5%). However, these trainees were largely unaware of predatory publishing and had limited and variable knowledge of different journal models or reputable journal practices. Consistent with prior literature that found that most authors who submitted to predatory journals had limited publications and few citations, the junior trainees in our study were more vulnerable to predatory publishing tactics [22,23]. Our study adds to the literature on predatory publishing by exploring the knowledge of predatory journal practices and publication practices of medical trainees in the developing medical education system of the UAE.

Our findings have important implications for curricular and educational reform. Although most trainees who had published chose open access journals (60.8%), very few were aware of Beall’s list, the Directory of Open Access Journals (DOAJ), the Think.Check.Submit initiative, or any other platform to assess target journal credibility. In fact, of trainees who reported receiving email invitations to submit manuscripts, 15% chose to submit to the unfamiliar (and possibly predatory) journal, mainly citing peer recommendations as the reason behind their decision. This observation suggests that the learning communities of our trainees can potentiate the impact of educational interventions and help spread awareness of predatory journal practices. Given the inconsistencies and concerns with individual checklists, it is more reliable for trainees to learn to identify characteristics of reputable journals and to employ due diligence in evaluating peer review processes and the practices of journals and publishers [31].

Many trainees reported pressures from the residency program to publish as another contributing factor to the decision submit to unsolicited requests from unfamiliar journals – posing significant risk for falling victim to predatory journals. Additionally, the overwhelming majority of respondents (close to 97%) cited that improving their candidacy for residency and fellowship acceptance was an important consideration influencing their decision to conduct research. This is consistent with other studies that have found that many authors willingly submit to predatory journals [12,32] and that program requirements and career advancement were the major drivers for participation in research by trainees [33]. In a study of general surgery residents, 80% reported that their motivation to conduct research was to ‘keep the program director happy’ [34]. This should raise concern and prompt educators to consider how to incorporate curricular requirements that facilitate scholarship and the spirit of scientific discovery without posing undue ‘check the box’ publication pressure on trainees. Requirements for research productivity need to be realistic, given the constraints of residency training, particularly in the international arena, where research support and mentorship may be limited and the prevalence of predatory practices is high.

Residents in our study also cited recommendation from faculty as an important factor in journal selection. Studies have shown that even senior researchers may unwittingly submit manuscripts to predatory journals [35]. In fact, some predatory journals have been identified on prestigious indices, including SCOPUS and Medline [32]. Understanding faculty knowledge of reputable journal practices is an important area for future study. Health professionals at all career levels should be encouraged to participate in awareness and education activities regarding reputable journal practices and campaigns. Based on our findings, we have redesigned our research methodology training to include sessions for both faculty and trainees to increase awareness of predatory journals, recognize reputable journal practices, and identify strategies to select the best journal for each study. We are also incorporating these educational goals into our evidence-based practice sessions.

We are cognizant, however, that awareness initiatives and educational reform alone will not deter potential authors from submitting to predatory publishers. Without a robust research infrastructure and explicit policies to deter submissions to predatory journals, the lower thresholds for manuscript acceptance and faster turnaround times of predatory journals will continue to appeal to authors and may sacrifice scientific standards and the credibility of the peer review process [36]. This is particularly pronounced in low and middle-income countries where academic institutions often pose comparable publication standards for promotion and advancement as industrialized nations, despite the significantly disparate access to the resources needed to meet them. A recent analysis in Turkey revealed that researchers submitted to lower quality journals to meet the minimum publication expectations for promotion and tenure [37]. A review of academic promotion criteria is necessary to ensure resources that are commensurate with institutional expectations and reduce ‘publish or perish’ pressures on faculty, which may fall prey to predatory practices.

Finally, our findings support the need for increased standardization and transparency of publisher practices and journal websites. Just as guidelines for conducting medical research studies have been established, such as the EQUATOR Network guidelines, publishers can provide guidelines for selecting reputable journals, and insist that their journals follow them [38].

Our study has several limitations. First, the small sample size from two teaching hospitals in one country limits generalizability. Next, the cross sectional design provides correlations; causality inferences cannot be made. Finally, inherent to any report of complex and evolving issues, such as reputable journal characteristics and motivation to publish, there are likely contributing factors that may not have been addressed in this study, including institutional culture, research infrastructure and mentorship availability and support.

## Conclusion

Open access journals benefit science by making high-quality research accessible to everyone. The exponential growth of predatory journals has made it increasingly difficult for authors and readers to distinguish these publications from legitimate, reputable journals. Our findings confirm that trainees in the UAE were largely unaware of reputable publication practices and were vulnerable to publishing in predatory journals. To maintain the credibility and integrity of the scientific process, policy and educational reform are necessary.

## Data Availability

The datasets generated and/or analyzed during the current study are not publicly available due to the institution’s policy to code and archive data in a central repository of the hospital in accordance to Abu Dhabi Department of Health regulations. Specific requests for remote access to de-identified data should be directed to clinicalresearch@seha.ae.
